# Mesenchymal phenotype predisposes lung cancer cells to impaired proliferation and redox stress in response to glutaminase inhibition

**DOI:** 10.1186/2049-3002-2-S1-P78

**Published:** 2014-05-28

**Authors:** Danielle Ulanet, Abhishek Jha, Kiley Couto, Sung Choe, Amanda Wang, Hin-Koon Woo, Mya Steadman, Byron DeLaBarre, Stefan Gross, Edward Driggers, Marion Dorsch, Jonathan Hurov

**Affiliations:** 1 Agios Pharmaceuticals, Cambridge, MA, USA

## Background

Recent work has highlighted glutaminase (GLS) as a key player in cancer cell metabolism, providing glutamine-derived carbon and nitrogen to pathways that support proliferation. There is significant interest in targeting GLS for cancer therapy however the gene is not frequently mutated or amplified in tumors. As a result, identification of tractable markers that predict GLS dependence is needed for translation of GLS inhibitors to the clinic.

## Materials and methods

Cells were engineered to overexpress an enzymatically active variant of GLS1 that does not bind the previously described GLS1 inhibitor, BPTES (bis-2-(5-phenylacetamido-1,2,4-thiadiazol-2-yl)ethyl sulfide), to validate the on-target effects of the drug and utility as a specific tool compound. A panel of NSCLC cells were screened with BPTES and the effects on proliferation were correlated with transcriptional/genetic markers.

## Results

Low E-cadherin and high vimentin expression, hallmarks of a mesenchymal phenotype, marked NSCLC cells that are particularly sensitive to inhibition of GLS1. Furthermore, lung cancer cells induced to undergo epithelial to mesenchymal transition (EMT) acquired sensitivity to the GLS inhibitor. Metabolic studies suggested that the mesenchymal cells have a reduced capacity for oxidative phosphorylation and increased susceptibility to oxidative stress, rendering them unable to cope with the perturbations induced by GLS inhibition.

## Conclusions

These findings elucidate selective metabolic dependencies of mesenchymal lung cancer cells and suggest novel pathways to target in this aggressive cancer type.

